# Enhanced Photocatalytic Activity toward Organic Pollutants Degradation and Mechanism Insight of Novel CQDs/Bi_2_O_2_CO_3_ Composite

**DOI:** 10.3390/nano8050330

**Published:** 2018-05-15

**Authors:** Zisheng Zhang, Shuanglong Lin, Xingang Li, Hong Li, Tong Zhang, Wenquan Cui

**Affiliations:** 1 School of Chemical Engineering and Technology, Tianjin University, Tianjin 300072, China; linshuanglong15@126.com (S.L.); lxg@tju.edu.cn (X.L.); lihong.tju@163.com (H.L.); 2 Department of Chemical & Biological Engineering, University of Ottawa, 161 Louis Pasteur St., Ottawa, ON K1N6N5, Canada; 3 National Engineering Research Center of Distillation Technology, Tianjin 300072, China; 4 College of Chemical Engineering, North China University of Science and Technology, Tangshan 063009, China; zhangt@126.com

**Keywords:** CQDs/Bi_2_O_2_CO_3_, up-conversion, flower microspheres, photocatalytic degradation

## Abstract

Novel carbon quantum dots (CQDs) modified with Bi_2_O_2_CO_3_ (CQDs/Bi_2_O_2_CO_3_) were prepared using a simple dynamic-adsorption precipitation method. X-ray diffractometry (XRD), transmission electron microscopy (TEM), energy dispersive X-ray spectroscopy (EDX), and scanning electron microscopy (SEM) were used to test the material composition, structure, and band structures of the as-prepared samples. Methylene blue (MB) and colorless phenol, as target organic pollutants, were used to evaluate the photocatalytic performance of the CQDs/Bi_2_O_2_CO_3_ hybrid materials under visible light irradiation. Experimental investigation shows that 2–5 nm CQDs were uniformly decorated on the surface of Bi_2_O_2_CO_3_; CQDs/Bi_2_O_2_CO_3_ possess an efficient photocatalytic performance, and the organic matter removal rate of methylene blue and phenol can reach up to 94.45% and 61.46% respectively, within 2 h. In addition, the degradation analysis of phenol by high performance liquid chromatography (HPLC) proved that there are no other impurities in the degradation process. Photoelectrochemical testing proved that the introduction of CQDs (electron acceptor) effectively suppresses the recombination of e^−^-h^+^, and promotes charge transfer. Quenching experiments and electron spin resonance (ESR) suggested that ·OH, h^+^, and ·O_2_^−^ were involved in the photocatalytic degradation process. These results suggested that the up-conversion function of CQDs could improve the electron transfer and light absorption ability of photocatalysts and ·O_2_^−^ formation. Furthermore, the up-conversion function of CQDs would help maintain photocatalytic stability. Finally, the photocatalytic degradation mechanism was proposed according to the above experimental result.

## 1. Introduction

In recent years, life and industrial sewage treatments have attracted increasing attention. Sewage is an important factor that affects environmental governance. Photocatalytic technology used for treating nocuous organic pollutants in water has recently received a lot of interest [1,2]. Pollutants can be effectively degraded via the generation of photoinduced electron-holes in photocatalysts under visible light irradiation. The above problems could be solved using semiconductor photocatalytic technology, which has been regarded as a promising green technology [3,4]. TiO_2_ has been a highly researched material because of its non-toxicity, chemical stability, commercial availability, high photoactivity, etc. [5,6,7] However, its practical application was limited by two main disadvantages: (1) poor visible light response ability, and (2) a low photogenerated charge separation efficiency [8]. Thus, many efforts have been made to explore new visible light photocatalytic materials which possess efficient photocatalytic activity.

In recent years, bismuth-based oxides which possess aurivillius-layered structures have been the focus of much research research, due to their hybridized valence band by O_2p_ and Bi_6s_ [9]. Some advantages of these photocatalysts are their good thermal stability, non-toxicity, etc. Some examples include BiVO_4_ [10,11,12], BiWO_6_ [13,14,15], BiOX (X = Br, Cl, I) [16,17,18], BiPO_4_ [19], and Bi_2_MoO_6_ [20]. Among these photocatalysts, the Bi_2_O_2_CO_3_ photocatalyst is a new type of semiconductor photocatalytic material which has a unique layer structure consisting of CO_3_^2−^ layers interwoven by [Bi_2_O_2_]^2+^ layers [21]. Thus, Bi_2_O_2_CO_3_ as a potential performance photocatalyst has gradually caught the attention of researchers. Thus far, the different morphological structures of Bi_2_O_2_CO_3_ have been studied, such as flower-like, sponge-like, porous ball, slice layer, etc. Various organic pollutants have also been used to investigate their photocatalytic performance [22,23]. However, because of its weak visible light response and wide band structure, modification research of Bi_2_O_2_CO_3_ is very important [24]. Hence, an effective way to improve the photocatalytic performance of Bi_2_O_2_CO_3_ may be to introduce a narrow band gap semiconductor, such as Bi_2_O_2_CO_3_/BiOI [25], Ag-AgBr/Bi_2_O_2_CO_3_ [26], Bi_2_O_2_CO_3_/Bi_2_S_3_ [27], etc.

Carbon quantum dots (CQDs), which are smaller than 10 nm in size, are well-dispersed spherical fluorescent nanocarbon materials. They gradually roused interest because of their easy functionalization, powerful chemical inertness, good photo-stability, excellent biocompatibility, and non-toxicity [28,29,30]. In addition, its photoluminescence (PL) up-conversion is a unique property [31]. Visible light emissions and shorter wavelength UV could be generated from near infrared (NIR) light radiation. The long wavelength of visible light makes CQDs a potential material for photocatalysis, solar cells, bioluminescence, etc., via the PL up-conversion effect [32,33,34].

CQDs can be either photoinduced electron donors or acceptors, due to their photoelectrochemical properties and their being richly fluorescent [28,35]. CQDs can either be used as a photocatalytic material or as functional components in composite photocatalyst designs to broaden the spectrum response range and promote the separation of photoinduced e^−^-h^+^ [32]. They can also inhibit semiconductor photolysis, such as in CQDs/Fe_2_O_3_ [36] and CQDs/ZnO [37].

In this research, we present a simple method to fabricate CQDs/Bi_2_O_2_CO_3_ composite with 3D flower micro-sphered structures. Structure and morphology characterizations, as well as the mechanism behind the efficiency promotion effect of CQDs/Bi_2_O_2_CO_3_, were carefully studied. The photocatalytic property of the CQDs/Bi_2_O_2_CO_3_ compound was investigated by removing methylene blue (MB) and colorless phenol, under simulated sunlight irradiation.

## 2. Experimental Methods

### 2.1. Photocatalyst Synthesis

All of the chemicals are analytical reagents without further purification.

#### 2.1.1. Synthesis of the 3D Flower-Like Bi_2_O_2_CO_3_

The 3D flower-like Bi_2_O_2_CO_3_ precursor was prepared by using the classic hydrothermal method. Using a classic course to synthesize the Bi_2_O_2_CO_3_ flower-like precursor, 0.003 mol of Bi(NO_3_)_3_·5H_2_O was dissolved in 20 mL of 1 M HNO_3_, and then 0.002 mol of citric acid was added. After 10 min stirring, NaOH was added to adjust the pH of the above reaction solution to 4.0–4.2. Then the reaction solution was transferred in a Teflon-lined, stainless steel autoclave, and maintained at 160 °C for 24 h. After natural cooling to room temperature, the prepared precursor was handled by repeated washing and centrifugation with ethanol and distilled water, and subsequently dried at 80 °C for 8 h.

#### 2.1.2. Preparation of Carbon Quantum Dots (CQDs)

A pair of graphite rods with a 7.5 cm separation were parallelly inserted into deionized water as the anode and counter-electrode (18.4 MΩ∙cm^−1^, 400 mL) [38]. A direct current (DC) supplies a 30 V static potential to the two electrodes. The solution then gradually became dark yellow, with corrosion being observed on the anode graphite rod. Finally, solution became a dark brown after a period of electrolytic process. The electrolyte was first filtered with filter paper. The filtrate was then centrifuged at 22,000 rpm for 30 min, which could remove the graphite particles and graphite oxide. The resultant solution is the ideal CQDs aqueous solution. (C_(CQDs)_ = 2.01 g/L)

#### 2.1.3. Preparation of CQD/Bi_2_O_2_CO_3_ Photocatalysts

CQD/Bi_2_O_2_CO_3_ composites were synthesized by a simple dynamic adsorption precipitation method. 0.3 g of Bi_2_O_2_CO_3_ photocatalyst were added to 50 mL of deionized water under ultrasonic treatment for 1 h. Next, a defined volume of CQD solution (10, 30, 50, 70 and 90 mL) was added to the above solution and stirred for 90 min at room temperature to create a clear dispersion of CQDs. The obtained samples were labeled as 10-CQDs/Bi_2_O_2_CO_3_, 30-CQDs/Bi_2_O_2_CO_3_, 50-CQDs/Bi_2_O_2_CO_3_, 70-CQDs/Bi_2_O_2_CO_3_, 90-CQDs/Bi_2_O_2_CO_3_, respectively. The CQD/Bi_2_O_2_CO_3_ photocatalysts were handled by repeated washing and centrifugation with ethanol and distilled water, and subsequently dried at 80 °C for 8 h.

### 2.2. Photocatalyst Characterization

The crystal structures and phase data of samples could be determined by X-ray diffractometry (XRD) (D/MAX2500 PC, Rigaku Corporation, Tokyo, Japan). The morphologies and composition of the samples could be investigated by transmission electron microscopy (TEM) (JEM-2010, JEOL Ltd., Akishima, Japan), energy dispersive X-ray spectroscopy (EDX) (s-4800, Hitachi, Chiyoda, Japan), and scanning electron microscopy (SEM) (s-4800, Hitachi, Chiyoda, Japan). The spectrofluorometer (f7000, Hitachi, Chiyoda, Japan) could be used to investigate the separation efficiency of photo-induced charge for powdered samples. The Perkin Elmer System 2000 infrared spectrometer provided the Fourier transform infrared (FTIR) spectra (Perkin Elmer, Shanghai, China), with KBr as the reference sample. The spin trapping electron spin resonance (ESR) measurements were performed on a Bruker JES FA200 ESR spectrometer (Oubeier, Beijing, China). The electrochemical and photoelectrochemical measurements were performed via a three-electrode quartz cell system. A CHI 660B electrochemical system (Shanghai Chenhua Instrument Corp., Shanghai, China) was used to record the photoelectrochemical results.

### 2.3. Photocatalytic Activity

The degradation rate of MB (or colorless phenol) was used to test the photocatalytic performance of CQDs/Bi_2_O_2_CO_3_ samples under the simulated sunlight (a 400 W metal halide lamp, λ > 400 nm, transmittance > 90%) in a multi-tube agitated reactor (XPA-7) (Xujiang, Nanjing, China). Thermostatic water provided cooling to control the reaction temperature (25 ± 2 °C). Firstly, 0.05 g of CQDs/Bi2O2CO3 powder was added to 50 mL of a 10 mg/L MB solution. Before light source irradiation, it was necessary to reach adsorption equilibrium for CQDs/Bi2O2CO3 in the absence of light about 30 min. During metal halide lamp irradiation, 3 mL reaction solution was measured out every 15 min in 120 min and stored in a dark environment. Finally, the collected reaction solutions were centrifuged for 6 min at 10,000 rpm, to separate the sample particles. A UV-vis spectrophotometer (UV-1901, Puxi, Beijing, China) was used to analyze the supernatant solutions (MB). (The determining wavelength: 400 nm < λ < 800 nm)

The degradation efficiency (%) was calculated as follows:

(1) $$\mathrm{Degradation}(\%)=\frac{C_{0}-C}{C_{0}}\times 100\%$$

where *C* and *C*_0_ were the *t* min and initial concentration of MB, respectively.

Additionally, blank and contrast tests were also performed. However, the high-performance liquid chromatography (HPLC) was used to analyze the concentration of phenol. The HPLC column was a 15 cm × 4.6 mm C18 column with a particle size of 5 μm. The mobile phase consisted of methanol/water = 70:30. The flow rate was 1 mL/min and column temperature of 35 °C.

### 2.4. Photoelectrocatalytic Activity

A photoelectrocatalytic test was used to evaluate the charge separation efficiency of the CQDs/Bi_2_O_2_CO_3_. In the test, CHI 660E Electrochemical Workstation (Shanghai Chenhua Instrument Corp., Shanghai, China) carried out all photoelectrochemical experiments results via a conventional three-electrode quartz cell system in a homemade quartz reactor (electrolyte: 0.1 M Na_2_SO_4_). A 500-W Xe lamp purchased (Beijing Changtuo Co. Ltd., Beijing, China) with a UV cut-off filter (420 nm) was used for light source. The prepared photoelectrode was used as a working electrode, while Pt was used as a counter electrode, and a saturated calomel electrode (SCE) was used as the reference electrode.

## 3. Results and Discussion

### 3.1. Catalyst Characterization

**Figure 1:**
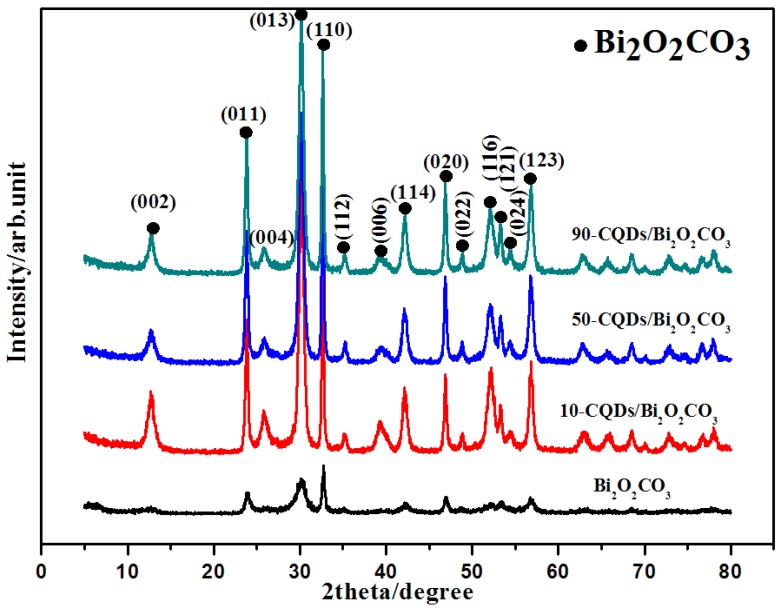
XRD patterns of the synthesized photocatalysts.

Figure 1 shows the XRD characteristic peak of the Bi_2_O_2_CO_3_ and different CQDs/Bi_2_O_2_CO_3_ composites, with various modified quantities of CQDs. The composition and crystalline properties of the photocatalysts can be identified using XRD. As shown, the curve of pure Bi_2_O_2_CO_3_ possesses many characteristic and distinct diffraction peaks, which perfectly match the diffraction peaks of tetragonal phase Bi_2_O_2_CO_3_ (JCPDS: 41-1488), suggesting satisfactory composition and purity. The main characteristic peak positions of samples appeared at 12.93°, 23.90°, 26.03°, 30.25°, 32.73°, 35.31°, 39.51°, 42.30°, 46.97°, 48.93°, 52.23°, 53.41°, 54.51° and 56.90°. This corresponds to the (002), (011), (004), (013), (110), (112), (006), (114), (020), (022), (116), (121), (024) and (123) crystal faces of Bi_2_O_2_CO_3_, respectively. In addition, the sharp and intense characteristic peaks suggested that the samples possessed good crystallinity. The XRD patterns of CQDs/Bi_2_O_2_CO_3_ photocatalysts were largely similar to those of Bi_2_O_2_CO_3_. The diffraction peaks of CQDs were not obvious, even at highly modified amounts, due to high dispersion and the small amount of the CQDs in the CQDs/Bi_2_O_2_CO_3_ composite.

An ultraviolet-visible (UV-vis) diffuse reflection spectrum can be used to measure the light absorption ability of photocatalysts. Additionally, the band gap energies of semiconductor photocatalysts could be calculated according to their electronic structure features on the UV-vis absorption curve. As shown in Figure S1a, less than 370 nm of UV light can be absorbed by the pure Bi_2_O_2_CO_3_ sample. The absorption ability of Bi_2_O_2_CO_3_ was weak in the visible region. After the introduction of CQDs, the light absorption ability of Bi_2_O_2_CO_3_ was markedly enhanced, and the absorption edges of the CQDs/Bi_2_O_2_CO_3_ exhibited a slight systematic red-shift. Furthermore, the light absorption intensity of CQDs/Bi_2_O_2_CO_3_ composites were obviously enhanced with increases of the CQD content. Additionally, the absorption spectrum of CQDs further confirms that the up-conversion function of CQDs could promote full spectrum absorption. Figure S1b shows the band gap energies of the prepared photocatalysts. The forbidden band of a crystalline semiconductor could be calculated using the following formula [39]:

(2) αhν=A(hν−Eg)n2

where α is the absorption coefficient, h is the Plank constant, ν is the light frequency, *E_g_* is the band gap and A is a constant [40]. 

The value of *n* is 1 or 4, depending on the photocatalyst type for a direct or indirect transition, respectively. Therefore, the *n* value of Bi_2_O_2_CO_3_ is 4 due to the indirect transition band gaps [41,42]. The plot of (αhν)^1/2^ versus the photon energy (hν) could calculated the band gap energy of Bi_2_O_2_CO_3_ and CQDs/Bi_2_O_2_CO_3_ through the intercept of the tangent to the *x*-axis. As shown in Figure S1b, the band gap of pure Bi_2_O_2_CO_3_ was approximately 3.4 eV. The above results indicated that CQDs/Bi_2_O_2_CO_3_ composites have significant visible light absorption ability, due to the deposition and up-conversion behavior of CQDs. The introduction of CQDs effectively promotes the charge-transfer between CQDs and Bi_2_O_2_CO_3_. It broadens the optical absorption edges and enhanced the optical absorption intensity of CQDs/Bi_2_O_2_CO_3_ composites. In summary, the up-conversion behavior of CQDs effectively improved the photocatalytic degradation ability of CQDs/Bi_2_O_2_CO_3_ photocatalysts under visible light irradiation.

Valence band X-ray photoelectron spectroscopy (VB XPS) could be used to calculate the valence band (VB) position of photocatalysts. As shown in Figure S2a, the valence band of Bi_2_O_2_CO_3_ is about 3.56 eV. According to the result of Figure S1b, the conduction band (CB) edge of Bi_2_O_2_CO_3_ should be located at 0.16 eV. This result is supported by the Mott-Schottky (MS) plots experiment (Figure S2b). The MS plots for Bi_2_O_2_CO_3_ indicate typical n-type semiconductors with a typical shape [43]. The conduction band is about 0.15 eV. The measured value was close to the calculated value. The above results could provide a strong theoretical basis for the discussion of the photocatalytic degradation mechanism.

**Figure 2:**
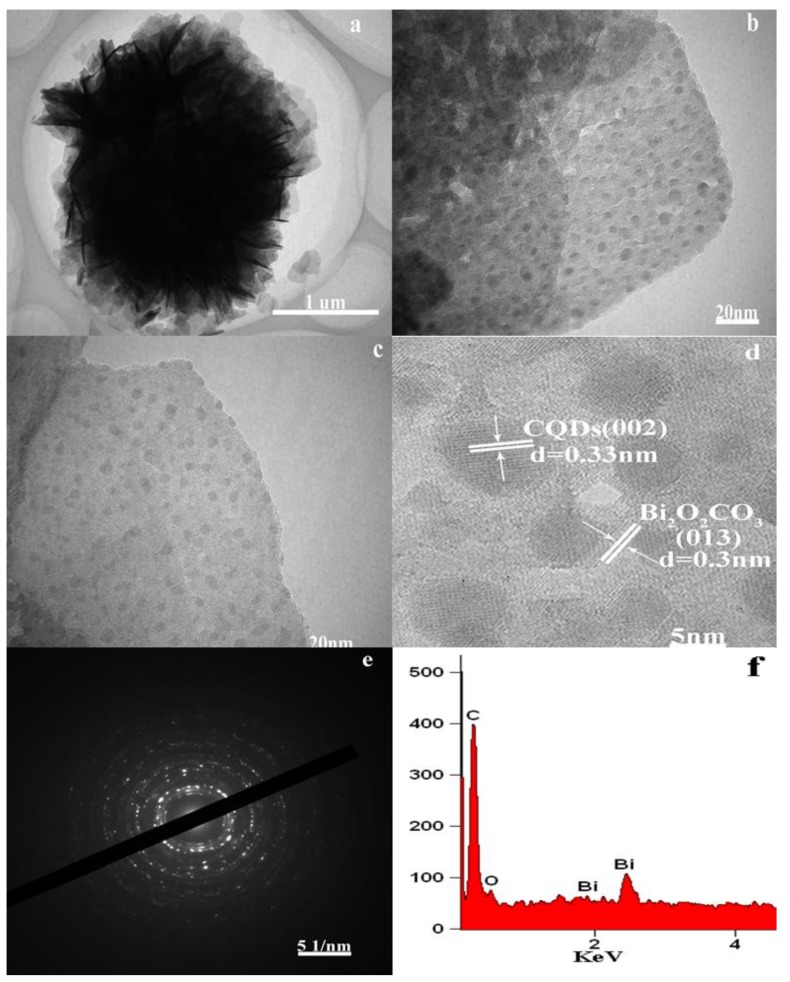
TEM images of as-prepared samples: (**a**) Bi_2_O_2_CO_3_; (**b**,**c**) CQDs/Bi_2_O_2_CO_3_; (**d**–**f**) HRTEM, SAED, EDX images of CQDs/Bi_2_O_2_CO_3_.

The TEM analysis was used to characterize the microstructure and morphology of Bi_2_O_2_CO_3_ and CQDs/Bi_2_O_2_CO_3_. Figure 2a shows the TEM image of the prepared Bi_2_O_2_CO_3_ sample. The average diameter of the flower-like microsphere structure was about 1 to 2 µm, which consists of 2D nanosheet structures. Figure 2b,c show the TEM images of CQDs/Bi_2_O_2_CO_3_ samples. It is obvious that a mass of CQDs are evenly attached to the surface of the Bi_2_O_2_CO_3_ “flower-like” nanosheets. The results demonstrated that the CQDs modified on the surface of Bi_2_O_2_CO_3_ were homodisperse, with a 2–5 nm spherical diameter. The modifications of CQDs can significantly increase the number of photocatalytic reaction sites and the surface roughness of the Bi_2_O_2_CO_3_ flower structure [44], thereby significantly improving the photocatalytic degradation efficiency. The HRTEM image (Figure 2d) showed that the interplanar spacing of CQDs and Bi_2_O_2_CO_3_ was 0.33 and 0.30 nm, corresponding to the (002) and (013) crystallographic planes, respectively. Figure 2e shows the selected-area electron diffraction (SAED) pattern of CQDs/Bi_2_O_2_CO_3_. The high-crystallinity of CQDs/Bi_2_O_2_CO_3_ photocatalysts was obvious according to the clear lattice fringe. The elemental composition of the CQDs/Bi_2_O_2_CO_3_ photocatalyst was estimated using EDS analysis. The EDS spectrum shown in Figure 2f revealed that the CQDs/Bi_2_O_2_CO_3_ composite photocatalyst contained only Bi, C, and O. The measured value of the C element was more than predicted in the theoretical value of C in Bi_2_O_2_CO_3_, which confirmed the existence of CQDs on the surface of Bi_2_O_2_CO_3_. The above results indicate the effective compounding of CQDs and Bi_2_O_2_CO_3_.

**Figure 3:**
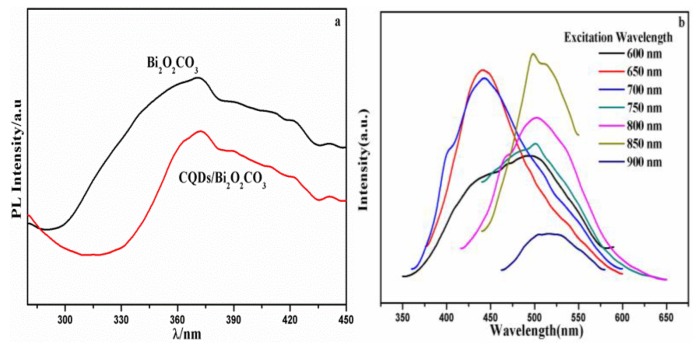
(**a**) Photoluminescence (PL) spectra of Bi_2_O_2_CO_3_ and CQDs/Bi_2_O_2_CO_3_; (**b**) PL curve of CQDs excited by different wavelengths of light.

Figure 3a shows the photoluminescence (PL) spectra of Bi_2_O_2_CO_3_ and CQDs/Bi_2_O_2_CO_3_. The PL spectra were dependent on the 260 nm excitation wavelengths. It is known that the recombination degree of the photogenerated e^−^-h^+^ could be reflected by the PL technique. Therefore, to a certain extent, the PL spectra could reflect the photocatalytic properties of semiconductors. Clearly, the shapes and peak positions of Bi_2_O_2_CO_3_ were similar to those of CQDs/Bi_2_O_2_CO_3_. However, the peak intensity of CQDs/Bi_2_O_2_CO_3_ decreased, suggesting the high separation efficiency of the carrier charge when CQDs are decorated on the surface of Bi_2_O_2_CO_3_. Figure 3b shows the fluorescence spectrum peaks of CQDs excited by long wavelengths. The up-conversion PL spectra show excitation-dependent PL feature spectra. With the excitation wavelengths increasing from 600 nm to 900 nm, the up-converted emission peaks exhibit a red shift from 430 nm to 520 nm. The above results show that CQDs have strong fluorescence up-conversion properties.

**Figure 4:**
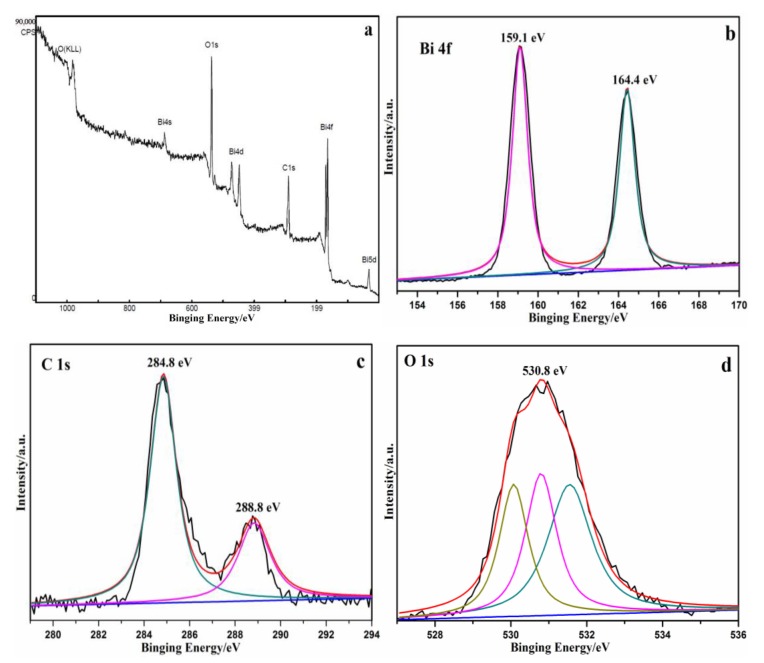
XPS characteristic peak of the CQDs/Bi_2_O_2_CO_3_ sample. (**a**) Survey of the sample; (**b**) Bi 4f; (**c**) C 1s; (**d**) O 1s.

The presence of CQDs in CQDs/Bi_2_O_2_CO_3_ photocatalysts could be further determined using a Fourier transform of the infrared (FTIR) spectra. As shown in Figure S3, by comparing the FTIR spectrums of CQDs, Bi_2_O_2_CO_3_ and CQDs/Bi_2_O_2_CO_3_, no obvious impurity absorption peaks were detected. For the CQDs/Bi_2_O_2_CO_3_ material, the stretching vibrations of the Bi-O in Bi_2_O_2_CO_3_ could be obtained according to the intensive peaks at about 560–850 cm^−1^. The stretching vibrations of C–O and C=O were assigned to the peaks at approximately 1400 cm^−1^ [45], respectively. The absorption band at approximately 1040 cm^−1^ attributes to C–C or C–O stretching vibrations. The existence of COO^−^ in CQDs could be proven by the presence of 1452 cm^−^^1^ absorption peaks [46]. The peaks at 1705–3454 cm^−1^ are due to the bending vibrations of water molecules absorbed on CQDs/Bi_2_O_2_CO_3_, and stretching vibrations of the –OH group. The peaks at about 2950 cm^−1^ are attributed to C–H stretching and rocking band vibrations, respectively [47]. All these results successfully prove the existence of CQDs and Bi_2_O_2_CO_3_, and suggest the combination and an interface interaction between CQDs and Bi_2_O_2_CO_3_. The XPS analysis (Figure 4) could be further used to prove the results.

The surface properties and chemical compositions of the samples could be determined by using an X-ray photoelectron spectrometer (XPS). Figure 4a displays the full survey spectrum of CQDs/Bi_2_O_2_CO_3_, which suggested the existence of oxygen (O 1s), carbon (C 1s) and bismuth (Bi 4f). The high-resolution XPS spectra of Bi 4f appeared at about 159.1 eV (Bi 4f_5/2_) and 164.4 eV (Bi 4f_7/2_) (Figure 4b), which belong to a crystal structure of Bi^3+^ [48]. In the narrow scan of the C 1s (Figure 4c), the main peak at 284.8 eV is ascribed to the C–C sp2-hybridizedcarbon of CQDs [49]. The characteristic peaks of the C 1s, which appeared at 288.8 eV, was due to the presence of C–O–C and/or C=O [50]. The peak of O 1s was shown in Figure 4d. The characteristic peak could be split into three peaks. The peaks at 531.5, 530.8 and 530.0 eV are ascribed to adsorption oxygen, hydroxyl oxygen, and lattice oxygen, respectively. The presence of CQDs in CQDs/Bi_2_O_2_CO_3_ samples could be confirmed according to the C 1s and O 1s patterns, which are consistent with the FTIR analysis results (Figure S4).

### 3.2. Photocatalytic Activity

**Figure 5:**
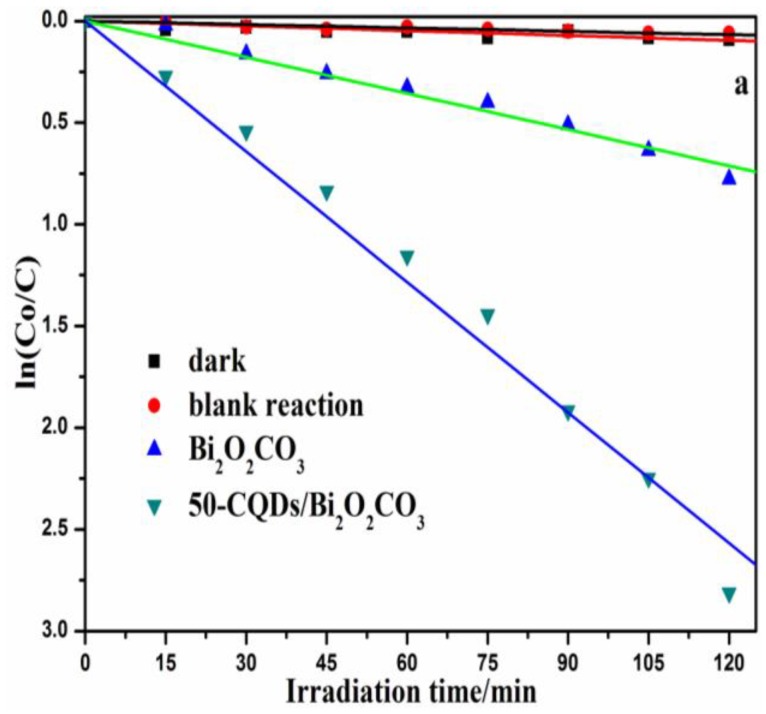

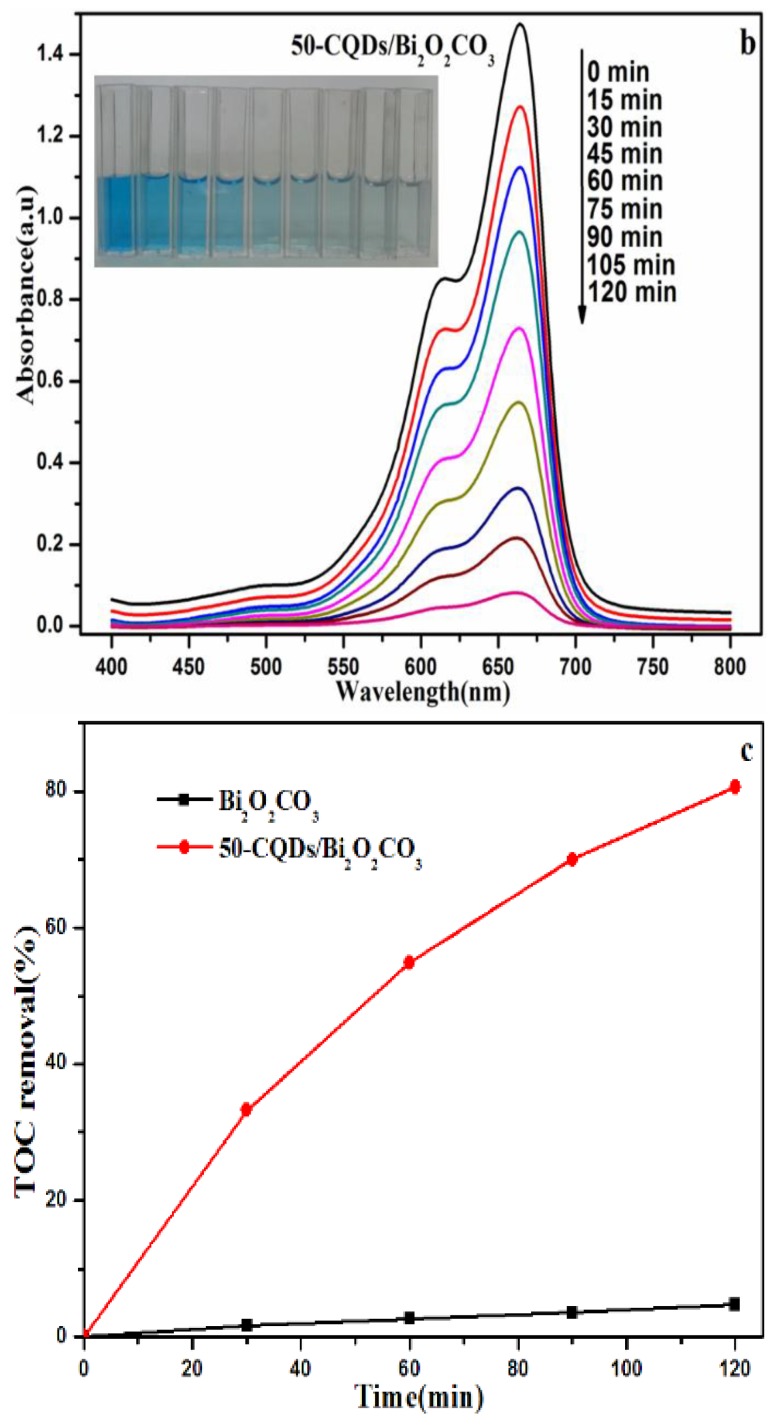
(**a**) Comparison of the photocatalytic degradation efficiency of MB by different photocatalysts (**b**) UV-vis spectral absorption changes of MB solution degraded by the 50-CQDs/Bi_2_O_2_CO_3_ composite; (**c**) TOC removal of MB.

MB was used as target pollutants to evaluate the photocatalytic degradation performance of the prepared samples under metal halide lamp irradiation. The 30 min absorption process was used to ensure the absorption-desorption balance between the photocatalyst and pollutants. Firstly, the degradation of MB by the composite without light irradiation, as well as the direct photolysis of MB without a photocatalyst, was investigated; the results are presented in Figure 5a. In the dark or without a photocatalyst, only a slight degradation of MB was observed. However, it is obvious that the photocatalytic property of Bi_2_O_2_CO_3_ improved significantly with the introduction of CQDs. 50-CQDs/Bi_2_O_2_CO_3_ showed the highest degradation efficiency (94.45%) within a 2 h irradiation, which was higher than that of Bi_2_O_2_CO_3_ (54.43%) for the same time period. CQDs could promote the photocatalytic process by improving the visible light responsiveness, or enhancing the charge separation efficiency of the system. Specifically, Bi_2_O_2_CO_3_ could be excited to generate the electrons-hole pairs by short wavelengths (ultraviolet light) by up-converting the long wavelength (visible light) via CQDs on the surface of Bi_2_O_2_CO_3_. Meanwhile, CQDs function as an excellent electron mediator and acceptor in CQDs/Bi_2_O_2_CO_3_ to effectively separate e^−^-h^+^ pairs, and improve degradation efficiency. The function mechanism was similar to that of previous studies [51,52,53]. Figure 5b shows the UV-vis absorption spectra temporal changes for MB solutions in the presence of 50-CQDs/Bi_2_O_2_CO_3_. It is obvious that the arresting characteristic peak (664 nm) weakened in intensity as the reaction time increased. Therefore, the final solution almost became colorless from the initial blue after approximately 120 min of irradiation, which further implies the complete destruction of the conjugated structure of MB. Figure 5c shows the total organic carbon (TOC) removal of MB as a function of reaction time. After 120 min illumination, the TOC removal by Bi_2_O_2_CO_3_ and 50-CQDs/Bi_2_O_2_CO_3_ were 4.67% and 80.64%, respectively. Additionally, High performance liquid chromatography (HPLC) was used to further verify the degradation process.

As is known, pseudo-first-order kinetics is suitable for the photocatalytic degradation of organic pollutants. The *k*_app_ values of CQDs/Bi_2_O_2_CO_3_ composites with different amounts of CQDs are shown in Figure S4. More CQDs resulted in higher kinetic constants for the CQDs/Bi_2_O_2_CO_3_, and the highest kinetic constant (0.0214 min^−1^) appeared when using 50 mL of CQDs. The kinetic constant did not increase with a further increase of CQDs, and even decreased sharply when 90 mL of CQDs was used. This may be due to the fact that when the CQD content is sufficiently high, there is an associated decrease in the number of available active sites, and thus, lower photoelectrocatalytic activity [54]. In such a situation, excess CQDs could act as recombination sites for electron-hole pairs. A suitable quantity of CQDs could effectively improve the synergetic effects of CQDs and Bi_2_O_2_CO_3_, which largely contributes to the separation of electron-hole pairs.

**Figure 6:**
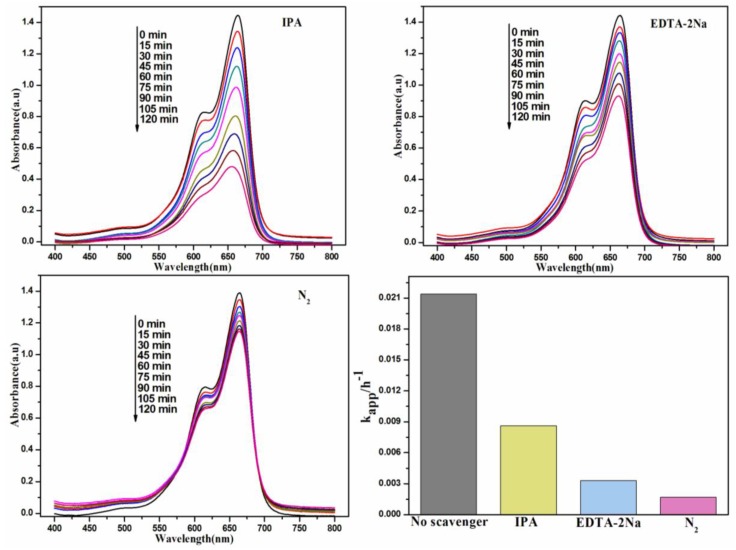
Different photocatalytic degradation performances after adding different quenching agents ((**a**) IPA, (**b**)EDTA-2Na, (**c**) N_2_); (**d**) The corresponding degradation kinetic constant.

In order to verify the role of active species in the organic pollutants degradation process over CQDs/Bi_2_O_2_CO_3_ hybrid materials in detail, quenching experiments were performed. As shown in Figure 6, isopropanol (IPA, 0.01 M) and ethylenediamine tetraacetic acid disodium (EDTA-2Na, 0.01 M) were used to quench the hydroxyl radicals (·OH) and holes (h^+^), Nitrogen (N_2_) was used as the superoxide radical (·O_2_^−^) scavenger. The addition of IPA, EDTA-2Na and N_2_ made the kinetic constants decrease from 0.0214 min^−1^ to 0.0086 min^−1^, 0.0033 min^−1^ and 0.0017 min^−1^, respectively. As the kinetic constants decreased, a greater effect was observed for the corresponding active species. Therefore, these above results suggested that the ·O_2_^−^, ·OH and h^+^ were involved in the photocatalytic degradation process. Moreover, the ·O_2_^−^ was the main active species in degradation process.

**Figure 7:**
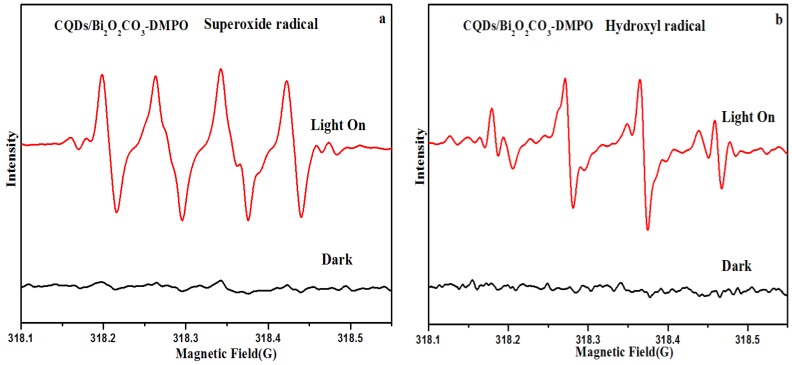
ESR spectra of radical adducts trapped by DMPO: (**a**) superoxide radical (·O_2_^−^); (**b**) hydroxyl radical (·OH).

To further confirm the generation of ·O_2_^−^ and ·OH species, the ESR spin-trap measurement based on CQDs/Bi_2_O_2_CO_3_ photocatalysts under dark and visible light irradiation was conducted. DMPO (5, 5-dimethyl-1-pyrroline *N*-oxide), a nitrone spin trapping reagent, was utilized to capture the superoxide (·O_2_^−^) and hydroxyl radicals (·OH). As shown in Figure 7a,b, negligible ESR signals were observed under dark conditions. However, the four-line characteristic ESR signal for DMPO-·O_2_^−^, a signal for the DMPO-·OH spin adduct were found under visible light irradiation. Furthermore, in the ESR spectra of DMPO-·OH for CQDs/Bi_2_O_2_CO_3_, four sets of antisymmetric peaks with intensities of 1:2:2:1 were observed, which are the characteristic signals of DMPO-·OH adducts. ESR demonstrates that ·O_2_^−^ and ·OH are the main active species of CQDs/Bi_2_O_2_CO_3_ in the photocatalytic degradation process under visible light illumination.

**Figure 8:**
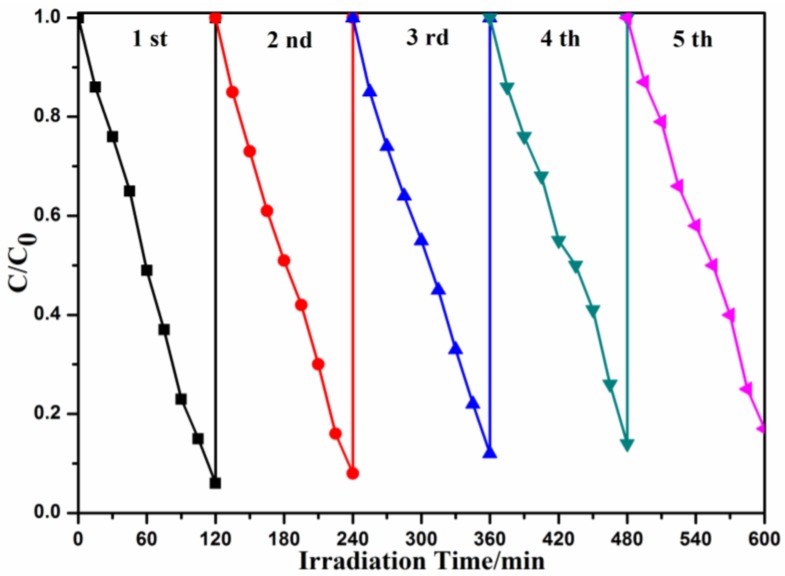
Cycling runs for the photocatalytic degradation of MB by CQDs/Bi_2_O_2_CO_3_ hybrid materials under visible light irradiation. (MB: 50 mL, 10 mg/L; CQDs/Bi_2_O_2_CO_3_: 1 g/L).

The stability of the photocatalysts is an important factor in its industrial application. Recycling reactions were used to evaluate the stability of the CQDs/Bi_2_O_2_CO_3_ samples. As shown in Figure 8, the degradation rate of CQDs/Bi_2_O_2_CO_3_ samples decrease from 94.45% (1st) to 82.91% (5th); the photocatalytic efficiency was only slightly lower. Considering the loss of catalyst in each cycling process and the test error, the experimental results imply that the CQDs/Bi_2_O_2_CO_3_ possessed good stability. This suggests that the oxygen vacancies of CQDs/Bi_2_O_2_CO_3_ could effectively be refreshed by the up-conversion effect of CQDs, and therefore, CQDs/Bi_2_O_2_CO_3_ possess good stability [55]. CQDs/Bi_2_O_2_CO_3_ materials have great potential and prospects for practical application in the future.

**Figure 9:**
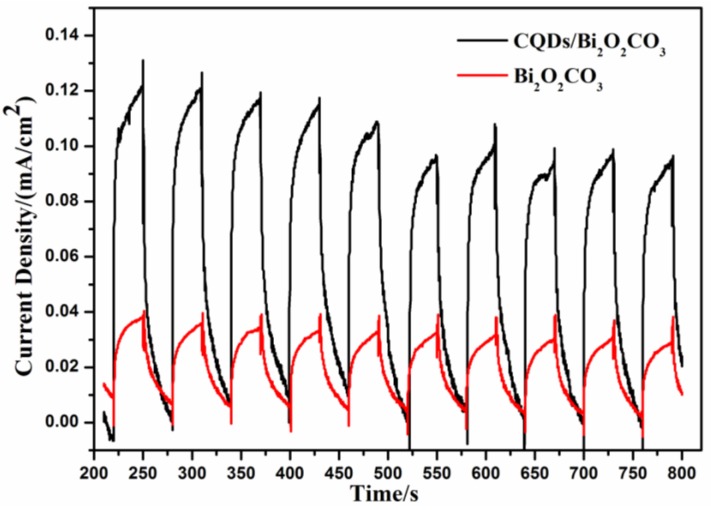
the photocurrent of Bi_2_O_2_CO_3_ and CQDs/Bi_2_O_2_CO_3_ under visible light.

**Figure 10:**
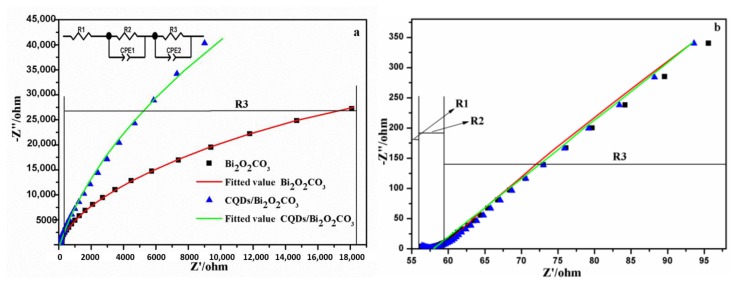
(**a**) Electrochemical impedance spectra (EIS) of Bi_2_O_2_CO_3_ and CQDs/Bi_2_O_2_CO_3_ under visible light; (**b**) The local amplification of (**a**).

The transient photocurrent response could be used to verify the separation efficiency of photoinduced charges in the samples, as shown in Figure 9. The photocurrent intensity of Bi_2_O_2_CO_3_ was 0.04 mA/cm^2^. The photocurrent intensity of CQDs/Bi_2_O_2_CO_3_ was higher than that of Bi_2_O_2_CO_3_ (0.11 mA/cm^2^). The results demonstrated that CQDs in CQDs/Bi_2_O_2_CO_3_ effectively improved the separation of the photogenesis charge, and increased the numbers of generated electrons [55], thus improving the degradation efficiency. These results were consistent with PL analysis (Figure 3); the electrochemical impedance spectra (EIS) (Figure 10) further confirmed this fact.

The most critical drawback in photocatalysis is the recombination process. EIS is quite a useful tool for investigating the charge transfer and recombination processes at the semiconductor/electrolyte interface [56]. Impedance spectra could be fitted by the ZsimpWin software according to the corresponding circuit. As shown in Figure 10a,b, the series resistance (R1) of the Pt counter electrode corresponds to the intercept on the real axis (high frequency). The charge-transfer resistance (R2) of the Pt counter electrode/electrolyte interface corresponds to the first semi-circle (high frequency). The charge-transfer resistance (R3) of the prepared samples anode/electrolyte interface corresponds to the second semi-circle (middle frequency) [57]. Compared with Bi_2_O_2_CO_3_, the smaller charge transfer resistance could be obtained from CQDs/Bi_2_O_2_CO_3_ using the smaller arc radius [58]. Thus, the performance of CQDs/Bi_2_O_2_CO_3_ contributed to the fast transfer and effective separation of e^−^-h^+^ pairs. The above results indicated that the introduction of CQDs could improve the electron transfer of photocatalysts and promote the photocatalytic property of CQDs/Bi_2_O_2_CO_3_.

**Figure 11:**
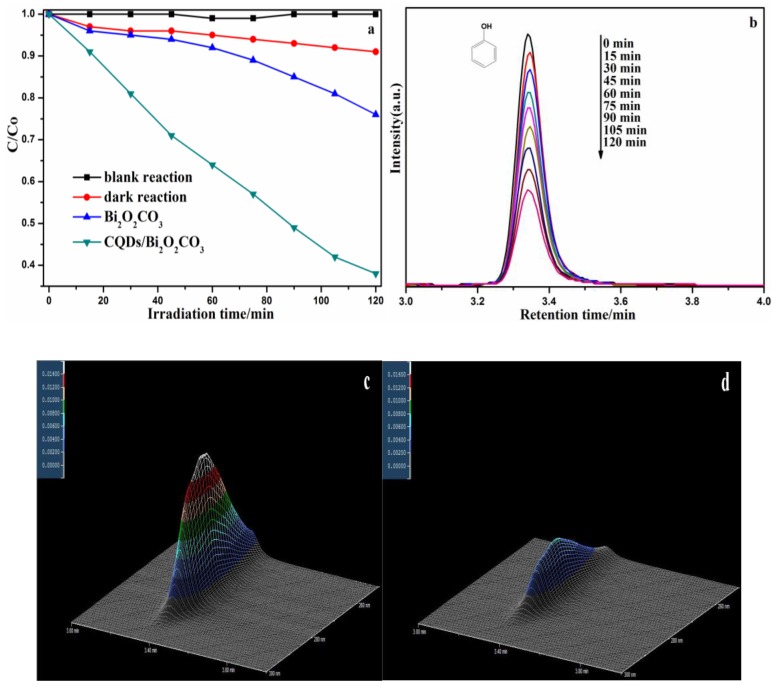
(**a**) The photocatalytic activity comparison of phenol degradation; (**b**) HPLC chromatograms of phenol solutions with CQDs/Bi_2_O_2_CO_3_ photocatalyst; (**c**,**d**) 3D HPLC spectra of phenol degradation at 0 min and at 120 min.

In order to prove the degradation ability of the CQDs/Bi_2_O_2_CO_3_ photocatalyst for organic pollutants, and explore the organic molecule changes in the degradation process, phenol was degraded as the target pollutant under visible light irradiation. Then, this was tested by using high performance liquid chromatography (HPLC). As shown in Figure 11a, the result revealed that the CQDs/Bi_2_O_2_CO_3_ sample had a higher photocatalytic property than pure Bi_2_O_2_CO_3_ for the phenol; 61.96% phenol was degraded for CQDs/Bi_2_O_2_CO_3_ within 120 min. As Figure 11b–d shows, the characteristic peak of phenol, located at 3.35 min retention time (RT), gradually weakens with increasing reaction time. No obvious impurity peak appeared in the degradation process. The results imply that phenol degradation was the mineralization process, and that CQDs could effectively promote the photocatalytic property of CQDs/Bi_2_O_2_CO_3_. Additionally, based on the degradation of two types of model organic pollutants (MB and phenol), the above results imply that the CQDs/Bi_2_O_2_CO_3_ has great potential for application in the treatment of organic pollutants.

**Figure 12:**
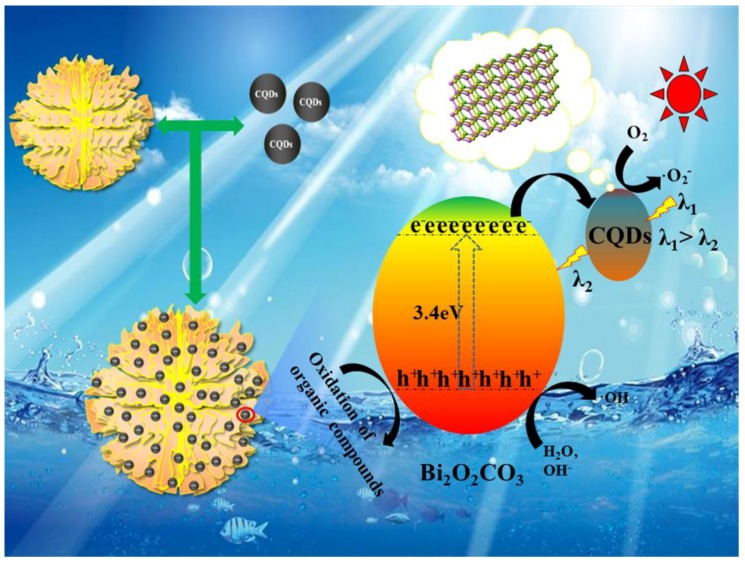
Schematic of the possible reaction mechanism for organic pollutants degradation by CQDs/Bi_2_O_2_CO_3_ under the simulated sunlight irradiation.

According to the characterization and experimental results above, it was found that the CQDs/Bi_2_O_2_CO_3_ exhibited an efficient photocatalytic degradation ability for organic contaminants. The obviously enhanced photocatalytic property of CQDs/Bi_2_O_2_CO_3_ may be attributed to its unique structure. The flower-like Bi_2_O_2_CO_3_ functions as a good stabilizer and substrate for CQDs, and the up-conversion effect of CQDs could improve the electron transfer and light absorption ability of photocatalysts and improve ·O_2_^−^ formation. The degradation schematic of CQDs/Bi_2_O_2_CO_3_ is presented in Figure 12. According to the literature and the experimental tests, the energy gap (*E_g_*) of Bi_2_O_2_CO_3_ is about 3.4 eV, and its valence band (VB) and conduction band (CB) potentials are 3.56 and 0.15 eV, respectively. Therefore, there is no obvious photocatalytic activity under visible light irradiation for Bi_2_O_2_CO_3_. However, CQDs, which possess the up-conversion effect and function as an excellent electron mediator and acceptor in CQDs/Bi_2_O_2_CO_3_, modify the surface of Bi_2_O_2_CO_3_, and enhance the visible light response of the photocatalyst due to the electronic coupling between conduction band states of Bi_2_O_2_CO_3_ and π states of CQDs [38]. As shown in Figure 12, the up-converted PL property of CQDs could take advantage of harnessing the full spectrum of sunlight. This, in turn, excites Bi_2_O_2_CO_3_ to form electron-hole pairs, thereby improving photocatalytic activity. Bi_2_O_2_CO_3_ could be excited to generate the electron-hole pairs by short wavelength ultraviolet light through up-converting the long wavelength visible light via CQDs on the surface of Bi_2_O_2_CO_3_. When CQDs/Bi_2_O_2_CO_3_ was irradiated by simulated sunlight, the electrons could be excited from the VB to the CB of Bi_2_O_2_CO_3_, leaving the holes on the VB of Bi_2_O_2_CO_3_. According to the PL spectra, CQDs facilitates the electron transfer from the CB of Bi_2_O_2_CO_3_ to oxygen across CQDs, retarding the recombination [59]. The electrons could then reduce the adsorbed O_2_ to ·O_2_^−^. Meanwhile the h^+^ on the VB of Bi_2_O_2_CO_3_ could react with the OH^−^ and/or H_2_O to produce ·OH, or a portion of the holes could also oxidize the organic pollutant directly [60]. The generated reactive oxygen species played an important role in the photocatalytic degradation process. These effects would guarantee the high photocatalytic activities of the CQDs modified Bi_2_O_2_CO_3_ samples.

## 4. Conclusions

In summary, CQDs modified Bi_2_O_2_CO_3_ (CQDs/Bi_2_O_2_CO_3_) was prepared by a simple dynamic adsorption precipitation method at room temperature. With CQDs approximately 2–5 nm in size evenly dispersed on the surface of Bi_2_O_2_CO_3_, CQDs/Bi_2_O_2_CO_3_ has efficient photocatalytic activity and long term stability under visible light. The photocatalytic degradation efficiency of methylene blue and phenol rate can reach up to 94.45% and 61.46% within 2 h, respectively. Photoelectrochemical testing proved that the introduction of CQDs (as electron acceptors) effectively suppresses the recombination of e^−^-h^+^ and promotes charge transfer. The quenching results suggested that ·O_2_^−^, h^+^ and ·OH were involved in the photocatalytic degradation process. These results demonstrated that the excellent photocatalytic performance for CQDs/Bi_2_O_2_CO_3_ can be attributed to the up-conversion effect of CQDs. The up-conversion effect of CQDs could improve ·O_2_^−^ formation and the electron transfer and light absorption ability of photocatalysts. Furthermore, the up-conversion effect of CQDs could help maintain photocatalytic stability. The above results can provide important inspiration for the development of other CQDs-based photocatalytic materials.

## Supplementary Materials

The following are available online at http://www.mdpi.com/2079-4991/8/5/330/s1, Figure S1: (a) UV-vis diffuse reflectance spectrum (DRS) and (b) the band gap energies (Eg) of Bi2O2CO3 and different CQDs/Bi2O2CO3 samples, Figure S2: (a) Valence band X-ray photoelectron spectroscopy (VB XPS) spectrum of Bi2O2CO3. (b) Mott-Schottky (MS) plots of Bi2O2CO3 photoelectrode, Figure S3: FTIR spectra of CQDs, Bi2O2CO3 and CQDs/Bi2O2CO3 samples, Figure S4: kinetic constant for the degradation of MB with the CQDs/Bi2O2CO3 composites prepared with different amounts of CQDs.
