# Supplementary material for: Enhanced Photocatalytic Activity toward Organic Pollutants Degradation and Mechanism Insight of Novel CQDs/Bi2O2CO3 Composite
Source: Nanomaterials (Basel). 2018 May 15;8(5):330. doi: 10.3390/nano8050330 (PMC5977344; doi:10.3390/nano8050330)
Supplement: Supplementary file 1 [file nanomaterials-08-00330-s001.pdf]

nanomaterials

Enhanced photocatalytic activity toward organic pollutants degradation and mechanism insight of novel CQDs/$Bi_2O_2CO_3$ composite

Zisheng Zhang [1,2,*], Shuanglong Lin [1], Xingang Li [1,3], Hong Li [1], Tong Zhang [4] and Wenquan Cui [4,*]

1 School of Chemical Engineering and Technology, Tianjin University, Tianjin 300072, China; linshuanglong15@126.com (S.L.); lxg@tju.edu.cn (X.L.); lihong.tju@163.com (H.L.)
2 College of Chemical Engineering, North China University of Science and Technology, Tangshan 063009, China
3 National Engineering Research Center of Distillation Technology, Tianjin 300072, China
4 Department of Chemical & Biological Engineering, University of Ottawa, 161 Louis Pasteur St., Ottawa K1N6N5, Canada; zhangt@126.com
* Correspondence: zzhang@uottawa.ca (Z.Z.); wkcui@163.com (W.C.); Tel.: +001-613-796-9771 (Z.Z.); +86-315-2592169 (W.C.)

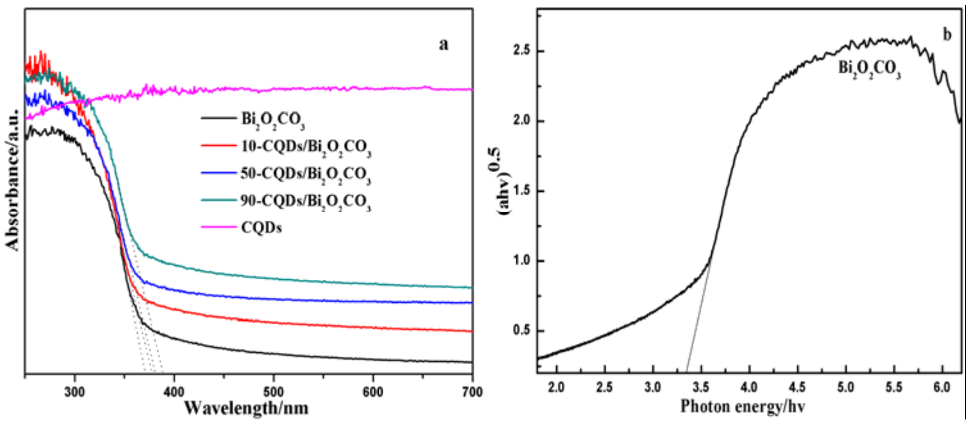

Figure S1. (a) UV-vis diffuse reflectance spectrum (DRS) and (b) the band gap energies (Eg) of $Bi_2O_2CO_3$ and different CQDs/$Bi_2O_2CO_3$ samples.

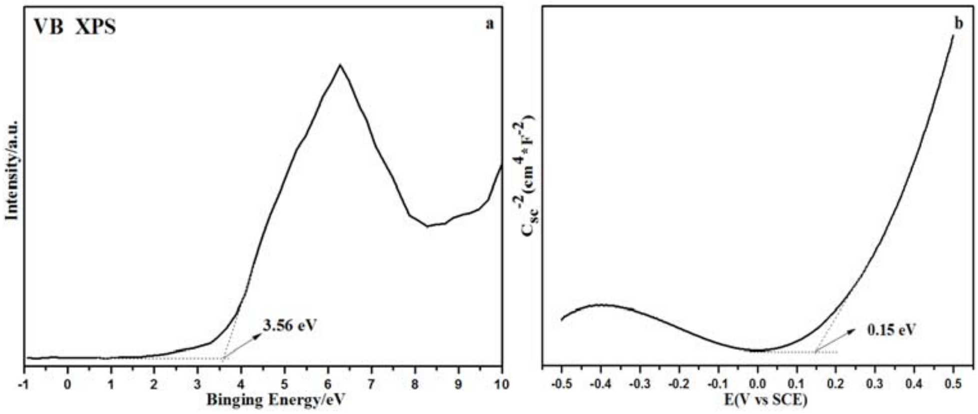

Figure S2. (a) Valence band X-ray photoelectron spectroscopy (VB XPS) spectrum of $Bi_2O_2CO_3$. (b) Mott-Schottky (MS) plots of $Bi_2O_2CO_3$ photoelectrode

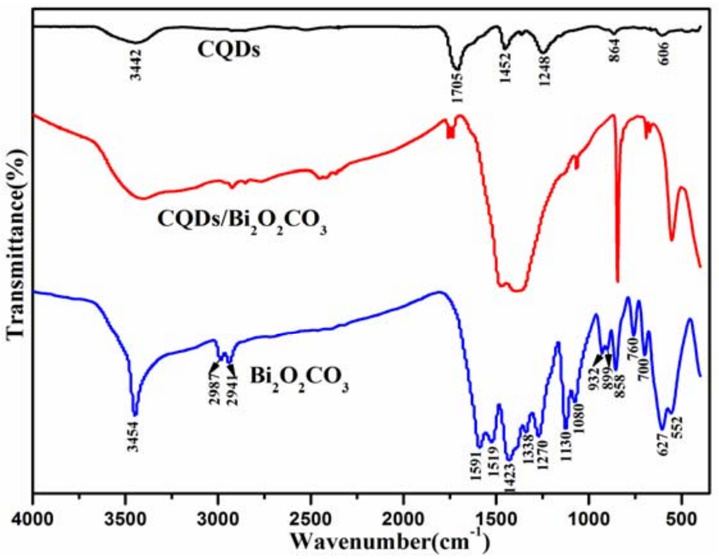

Figure S3. FT-IR spectra of CQDs, $Bi_2O_2CO_3$ and CQDs/$Bi_2O_2CO_3$ samples.

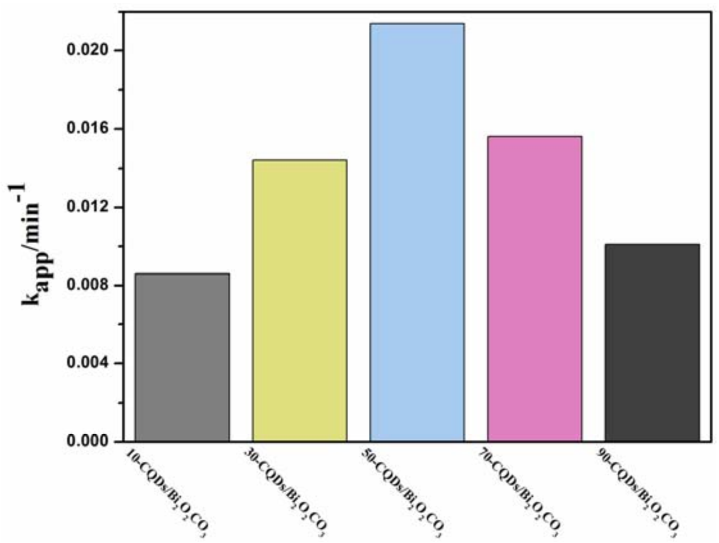

Figure S4. kinetic constant for the degradation of MB with the CQDs/$Bi_2O_2CO_3$ composites prepared with different amounts of CQDs
